# Dynamics and biodiversity of microbial community among seasons in Shanxi mature vinegar fermentation by semisolid-solid process

**DOI:** 10.1128/spectrum.00231-24

**Published:** 2024-11-13

**Authors:** Chen Li, Rong Kou, Yingying Jia, Xiaojun Fan, Ying Shi, Qihe Chen

**Affiliations:** 1 School of Life Science, Shanxi University, Taiyuan, China; 2 College of Environmental Science and Engineering, Taiyuan University of Technology, Jinzhong, China; 3 Shanxi Xinghuacun Fenjiu Group Co., Ltd., Fenyang, China; 4 Department of Food Science and Nutrition, Zhejiang University, Hangzhou, China; University of Mississippi, University, Mississippi, USA

**Keywords:** Shanxi mature vinegar, semisolid-solid process, microbiota assembly, seasonal characteristics, co-occurrence networks, UPGMA cluster analysis

## Abstract

The dynamic succession and seasonal characteristics of microbiota throughout the Shanxi mature vinegar (SMV) fermentation by the semisolid-solid process were explored using high-throughput sequencing techniques. The results showed that the richness and diversity of fungi were higher than those of bacteria in a complete seasonal SMV fermentation cycle, and the microbial community was dominated by 11 taxa of bacteria and 16 taxa of fungi. In all four seasons, lactic acid bacteria and acetic acid bacteria were the dominant bacteria, while the dominant fungi varied. *Saccharomyces* and *Pichia* played an important role in spring. *Aspergillus* and *Issatchenkia* were enriched in the summer. *Kazachstania* was the dominant microorganism in autumn. While *Mesenteroides* and *Meyerozyma* were enriched in winter. Unweighted pair group method with arithmetic mean (UPGMA) cluster analysis demonstrated that seasonality had a more decisive impact on microbiota composition than the fermentation stage within a season, and the microbiota structure in summer was significantly different from that in the other three seasons. Combined with the highest operational taxonomic units (OTUs) percentage (37%) of summer fungi in the Venn diagrams, it is speculated that the specific fungi may be the root cause for the relatively low SMV quality in summer. This work provided critical insights into the dynamic succession of the microbial community in SMV fermentation from a seasonality view, and the results could enrich our understanding of the microbiota involved in SMV fermentation and guide process control.

**IMPORTANCE:**

Understanding the changes in microbial communities across different seasons is crucial for ensuring the quality of Shanxi mature vinegar (SMV) by the semisolid-solid process. In a complete seasonal cycle, the richness and diversity of fungi were higher than those of bacteria. The microbial community in summer fermentation was significantly different compared to the other three seasons. For example, the dominant microorganisms such as *Acetobacter* and *Lactobacillus* decreased in summer. Screening or modifying this group of bacteria to enhance their tolerance to high fermentation temperature is an approach to improve industrial SMV fermentation. Through co-occurrence network analysis, eight highly connected genera were identified, which may play important roles in ecosystem stability. These results also lay a theoretical foundation for the further development of multi-microbial co-fermentation. This work provides an understanding of SMV fermentation from a seasonal perspective and offers new guidance for the process control of grain vinegar brewing.

## INTRODUCTION

Vinegar is an important fermented food used as an acidic condiment or preservative worldwide. Shanxi mature vinegar (SMV), with a history of over 3,000 years, is one of the most famous vinegars in China (1). Beyond its distinctive taste, SMV is known for its richness in active compounds such as flavonoids and tetramethylpyrazine (2–4). Since SMV gained recognition as a national geographic indication product of China in 2004, the market demand for SMV has steadily increased. However, the fermentation process of SMV is complex and its quality is influenced by various factors, resulting in unstable production and quality (flavor and physicochemical properties), which restricts the development of the SMV industry.

SMV is fermented using sorghum, *daqu* (a mixed culture starter containing various microorganisms), chaff husk, and wheat bran as raw material. The fermentation process of SMV consists of three main stages, namely starch saccharification (Sac), alcohol fermentation (AF), and acetic acid fermentation (AAF) (5). Sac and AF typically occur concurrently after steamed sorghum is blended with *daqu* and water. Once AF is complete, wheat bran, rice hull, and old Pei (the acetic acid fermented product from the previous batch, used as a vinegar starter) are added to initiate AAF (6). Based on water content, AF can be classified into liquid, semisolid, and solid fermentation processes, while AAF is always a solid fermentation process. Although solid-solid state fermentation is the main method for producing SMV, the semisolid-solid fermentation process has recently gained significant attention. This is because, compared to solid fermentation for AF, semisolid fermentation increases both sugar availability and alcohol production (7). Due to the open fermentation technique, numerous microbiotas coexist and promote the fermentation, playing an important role in the yield and quality of SMV. Microbial community composition and succession are greatly affected by multiple factors, for instance, the starter *daqu*, the fermentation process, and environmental factors (8). SMV brewing process is driven by a multispecies community and these microbiomes can present strong repeatable seasonal patterns like most other fermented foods, such as cheese (9), grape wine (10), and Chinese Luzhou-flavor liquor (11). It has been shown that the composition of the microbial community varies with different seasons in strongly human-controlled fermentation systems, such as the fermentation of Chinese Baijiu (12). In practical production, the fermentation of Zhejiang rosy vinegar is performed from May to September (13). For SMV, its yield and quality produced in summer are lower than those brewed in other seasons. In addition, the spoilage of vinegar brewing sometimes occurs on hot summer days (14). Current research mainly focuses on microbial changes during vinegar fermentation from the perspective of the brewing process. It should be noted that both the brewing process and seasons may lead to variations in microbial community composition. Our recent work has explored the differences in microbial structure during SMV production by solid-solid fermentation in summer and winter (14). Nonetheless, so far, the composition and dynamic succession of the microbial community by semisolid-solid fermentation on a whole seasonal scale is still far from clear, which hinders the final quality control of SMV in different seasons.

It is difficult to isolate the vast majority of microorganisms from natural systems due to the limitation of pure-culture techniques. With the emergence of culture-independent sequencing methods, the assembly and diversity of microbial communities in complex systems, such as human microbiome and fermented food, have been discovered (15, 16). Moreover, co-occurrence patterns provide a new perspective for exploring the community structure and function in a complex microbial environment. Microbial community characteristics and their co-occurrence patterns are variform and important in understanding microbiota assembly, thus offering critical insights into potential interaction networks, and revealing niche spaces shared by community members (17). Microbial co-occurrence patterns in different systems, including the ocean, soil, human gut, and fermented food, have been extensively studied, which is expected to provide a theoretical basis for investigating the horizontal gene transfer and assembly mechanisms of microbial communities (18, 19).

In this study, Ion S5 XL high-throughput sequencing technology and statistical methods were employed for the first time to describe the assembly and succession of SMV microbiota produced by semisolid-solid fermentation throughout the four seasons. This work helps us better understand the influence of seasonal changes on microbiota succession and interaction and provides a reference for developing strategies to improve the process control of SMV fermentation.

## MATERIALS AND METHODS

### Sample collection

Samples were collected throughout the entire SMV fermentation process in all four seasons (from May 2019 to February 2020) from a famous manufacturer located in Qingxu, Shanxi, China. For the semisolid-solid fermentation of SMV, the Sac stage, AF stage, and AAF stage were conducted for 3, 12, and 13 days, respectively. One sample of Sac stage (2 days of Sac, Sac 2), four samples of AF stage (1, 4, 8, and 12 days of AF, namely, AF1, AF4, AF8, and AF12), and four samples of AAF stage (2, 5, 7, and 10 days of AAF, namely, AAF2, AAF5, AAF7, and AAF10) were periodically collected. Semisolid mashes were collected from the surface, center, and bottom of the fermentation broth. Three random vinegar *Pei* samples were collected at a depth of 30 cm from the surface of the fermentation pit, and mixed uniformly as the samples of the fermentation stage. Samples (about 100 g) were collected and immediately stored in an ice box. All samples were kept at −20°C for further analysis.

### Genomic DNA extraction and PCR amplification

The genomic DNA was extracted using the CTAB method as described previously (20). Two pairs of specific primers were used to amplify the V4 region of bacterial 16S rRNA genes and fungal ITS2 region, respectively. The primers for the V4 region of bacterial 16S rRNA genes were 515F (5′-GTGCCAGCMGCCGCGGTAA-3′) and 806R (5′-GGACTACHVGGGTWTCTAAT-3′). The primers for the fungal ITS2 region were ITS3-2024F (5′-GCATCGATGAAGAACGCAGC-3′) and ITS4-2409R (5′-TCCTCCGCTTATTGATATGC-3′), respectively. To ensure amplification efficiency and accuracy, a high-fidelity PCR mix and enzyme (New England Biolabs) were used for PCR. The PCR products were visualized by 2% agarose gel electrophoresis and recovered using a gel extraction kit (Thermo Fisher Scientific).

### Sequencing analysis

The library was built using the Ion Plus Fragment Library Kit (Thermo Fisher Scientific). After quantification and library detection, IonS5 XL (Thermo Fisher Scientific) was used for on-machine sequencing. When obtaining the deplaning data, the low-quality reads were removed using cutadapt (21). Then, each sample data was extracted from the reads obtained by barcode. The barcode and primer sequences were removed for preliminary quality control to obtain raw reads (22). Compared with the community annotation database, the chimeric sequences in raw reads were removed to obtain clean reads.

### Microbial diversity and statistical analysis

The trimmed sequences were clustered into operational taxonomic units (OTUs) with 97% sequence similarity using Uparse (23). Then the species annotation of bacterial OTU sequences was analyzed by Mothur software and the SSU rRNA database of SILVA, and the taxonomic information was obtained (24). Blast method in QIIME (Version 1.9.1) (25) and Unit (v7.2) database (26) were used to analyze the species annotation of fungal OTUs sequences. Chao1, Shannon, Simpson, ACE, and Goods-coverage index via QIIME (Version 1.9.1) were calculated and beta diversity analysis was performed using Unifrac distances and visualized via principal coordinates analysis (PCoA). To determine differences within and among seasons, UPGMA was used to test significant differences among sample groups. The microorganism with significant differences in each group was determined using Linear discriminant analysis (LDA) effect size (LEfSe). Co-occurrence analysis was performed by calculating Spearman’s correlation coefficient between the predominant genera (with a relative abundance above 1% in at least one season). Networks were visualized using the network visualization software Gephi.

### Nucleotide sequence accession number

The sequences of bacterial 16S rRNA genes and fungal ITS2 regions were publicly available in the NCBI Sequence Read Archive under accession numbers SRP291834 and SRP294293, respectively.

## RESULTS AND DISCUSSION

### Alpha and beta diversity analysis of SMV fermentation

A total of 3,021,093 raw reads from the V4 region of bacterial 16S rRNA gene sequences and 2,724,560 raw reads from fungal ITS2 sequences were obtained from 36 samples throughout the SMV fermentation process in all four seasons. After filtering the low-quality reads and removing chimera sequences, an average of 79,442 bacterial clean reads and 73,885 fungal clean reads, respectively, were generated for further study (Table S1).

The alpha diversity indices, including Shannon, Simpson, Chao1, ACE, and Goods coverage, indicated that the diversity and richness of microbiota varied throughout the fermentation process in all four seasons (Table S2). Goods coverage of each sample was greater than 0.99 and all the rarefaction curves showed a clear-cut asymptotic plateau, which demonstrated that sequencing provided sufficient coverage in terms of classification diversity (Fig. S1). The Chao1 index of bacteria in winter was higher than that in spring during the fermentation process except for AF1, and it was worth noting that the bacterial diversity reached the highest in AF1 among all four seasons. In contrast, the Chao1 index of fungi in winter was lower than that in spring throughout the fermentation process, indicating a possible complementary effect between bacterial and fungal communities (Table S2). Additionally, as indicated by the Chao 1 and Shannon indices (Table S2), the richness and diversity of fungi were higher than those of bacteria at the same fermentation stage in all four seasons. This finding is consistent with the observations made during the fermentation of Sichuan Baoning vinegar (27). It should be noted that Sichuan Baoning vinegar differs from SMV. For example, Sichuan Baoning vinegar uses wheat bran as the principal ingredient, and Sac, AF, and AAF all occur simultaneously in the same fermentation pond, with a fermentation period of 30 days (27).

**Fig 1:**
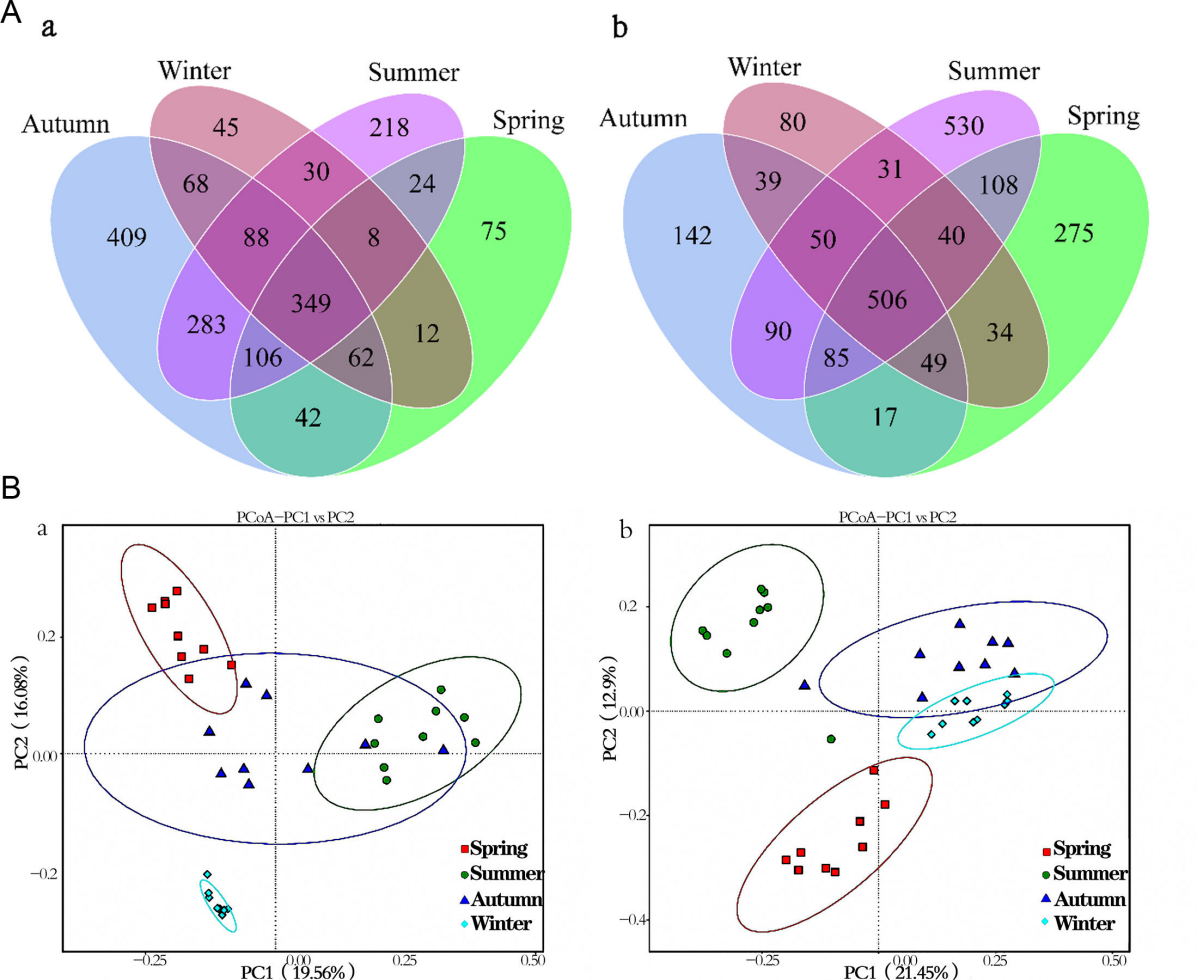
Venn diagrams (**A**) and PCoA analysis (**B**) of bacterial (**a**) and fungal (**b**) communities of SMV fermentation process across seasons.

Venn diagrams showed shared and unique OTUs of bacterial and fungal communities among different seasons (Fig. 1A). The shared OTUs in all four seasons accounted for 19% and 24% of total bacterial and fungal OTUs, respectively, suggesting a varied microbiota across seasons. The fungal components were richer than those of bacteria in a complete seasonal cycle (1,819 bacterial OTUs and 2,076 fungal OTUs). The bacterial OTUs in autumn were the most unique, accounting for 29% of the total, while the fungal OTUs in summer were the most unique, accounting for 37% of the total. The high-abundance microbiota could affect the stability of SMV fermentation, and microbial diversity might play an important role in stabilizing the fermentation process and enriching the metabolites.

PCoA based on UniFrac distances was also used to illuminate the clustering of SMV samples in different seasons (Fig. 1B). The results revealed that the first two components explained 35.64% and 34.35% of the bacterial and fungal variability, respectively. The clustering of microbiota was closer in winter samples, particularly for the bacterial community, suggesting that microbes at different fermentation stages in winter were more similar compared to the other three seasons. The sample distribution in autumn is wider, indicating that the changes in the microbial community in autumn may be greater.

**Fig 2:**
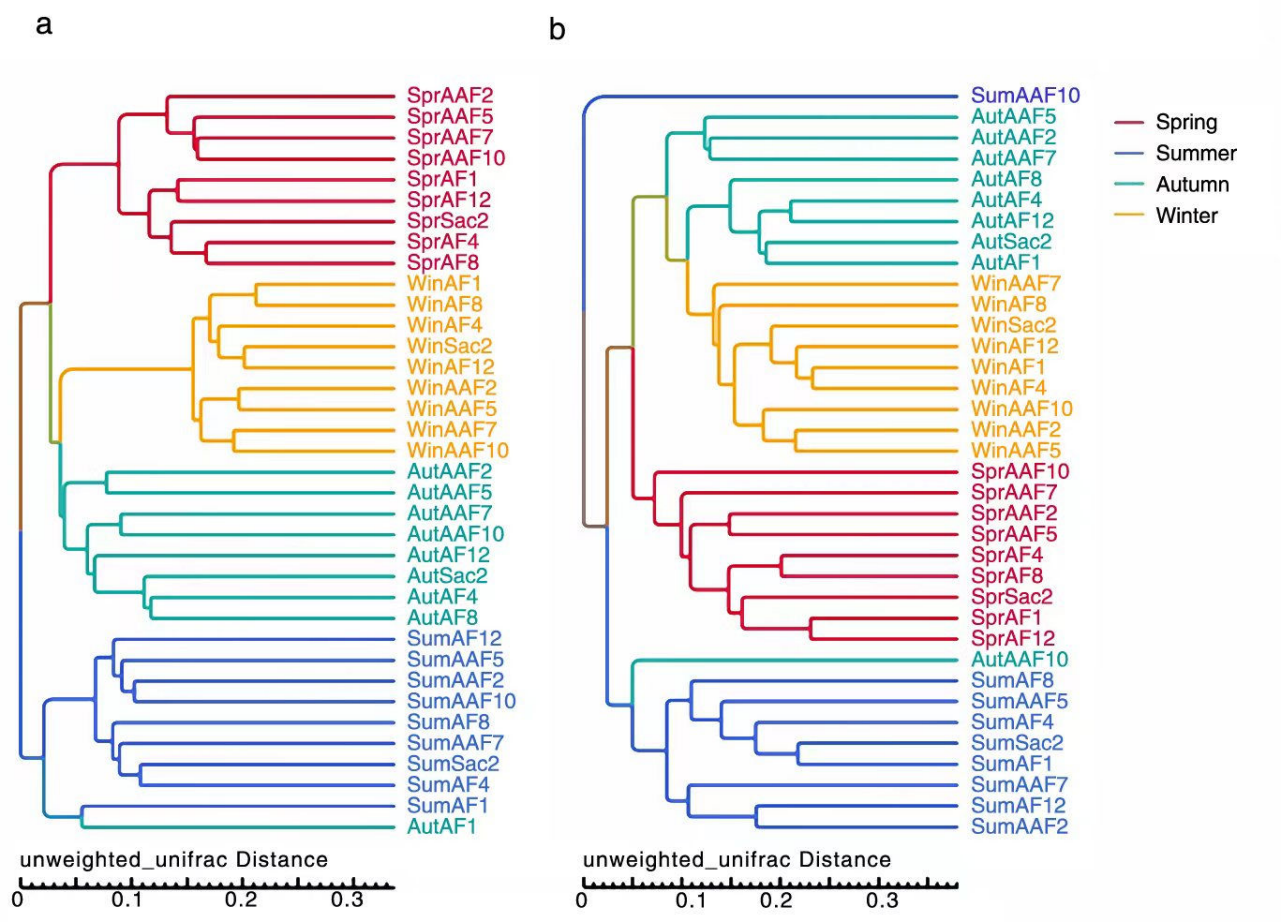
UPGMA clustering tree analysis of bacterial (**a**) and fungal (**b**) communities of SMV fermentation process across four seasons.

Clustering analysis was carried out using UPGMA to further evaluate the similarities and dissimilarities of microbiota within SMV samples (Fig. 2). The microbial community of SMV was strongly influenced by seasonality, as all samples from the same season gather together, except for AF1 and AAF10 samples in autumn and AAF10 samples in summer. There were significant phylogenetic differences between samples of the same fermentation stage in different seasons. This phenomenon indicated that seasonality even played a more decisive role in the microbiota composition of SMV than fermentation stages within the season. Furthermore, significant phylogenetic differences existed throughout the SMV fermentation process between summer and the other three seasons, which could be due to the high temperature in summer for SMV fermentation, resulting in a significantly different composition of the microbiota.

The marked increase in summer temperature, mainly caused by the greenhouse effect, poses a serious challenge for cereal vinegar fermentation and other traditional fermentation industries, as high temperatures can lead to a significant decrease in fermentation rate and efficiency (28). Usually, vinegar production is operated at 30°C. High temperatures can inhibit the growth of acid-producing bacteria, leading to reduced fermentation efficiency and potentially causing fermentation failure. Due to this reason, most Chinese distilleries and some cereal vinegar manufacturers stop production during the high-temperature period in summer (29). In this study, UPGMA clustering analysis showed significant differences in summer microbiota compared to the other three seasons. Shanxi province (34"35"−40"45" north latitude and 110"15"−114"32" east longitude) is located in north China. The weather in Shanxi is quite diverse throughout the year. In the summer, the temperature can reach up to 38°C. The high temperature decreases the abundance of acid-producing bacteria, which in turn reduces acid production and increases the risk of contamination. Combined with the high OTUs (37%) of summer fungi in the Venn diagrams, it can be inferred that the unique fungi in summer may be the root cause for the difficulty in controlling the summer SMV process and the relatively low quality.

### The taxonomic assembly of SMV fermentation on seasonal scales

To explore the dynamic changes of microbiota during SMV fermentation, the effective tags were classified into different taxonomic levels (phylum, class, order, family, and genus level) (Table 1). The bacterial community showed greater diversity than the fungal community at the phylum level in all seasons. Four dominant phyla of bacteria and three dominant phyla of fungi (average relative abundance ≥1%) were detected from SMV fermentation samples across four seasons (Fig. S2). Firmicutes dominated absolutely in all the fermentation stages across seasons, with an average relative abundance of above 88%. As the most dominant fungi, Ascomycota was widely distributed in vinegar samples, but its relative abundance declined rapidly in the AAF stage of SMV due to the fermentation procession and the synchronously changing fermentation environments. However, the relative abundance of Proteobacteria and Basidiomycota increased markedly in the AAF stage, and these microorganisms showed a clear division of labor in prophase and anaphase of SMV fermentation. For example, the abundance of Proteobacteria was increased in the anaphase of the AAF stage.

**TABLE 1:** The microbial composition of SMV brewing samples at different taxonomic levels across seasons

Classify	Bacteria				Fungi			
	Spring	Summer	Autumn	Winter	Spring	Summer	Autumn	Winter
Phylum	14	29	29	15	12	14	9	8
Class	23	48	46	22	27	39	23	25
Order	54	88	97	49	57	83	51	47
Family	96	152	184	84	118	166	106	91
Genus	165	287	377	163	190	257	165	138

**Fig 3:**
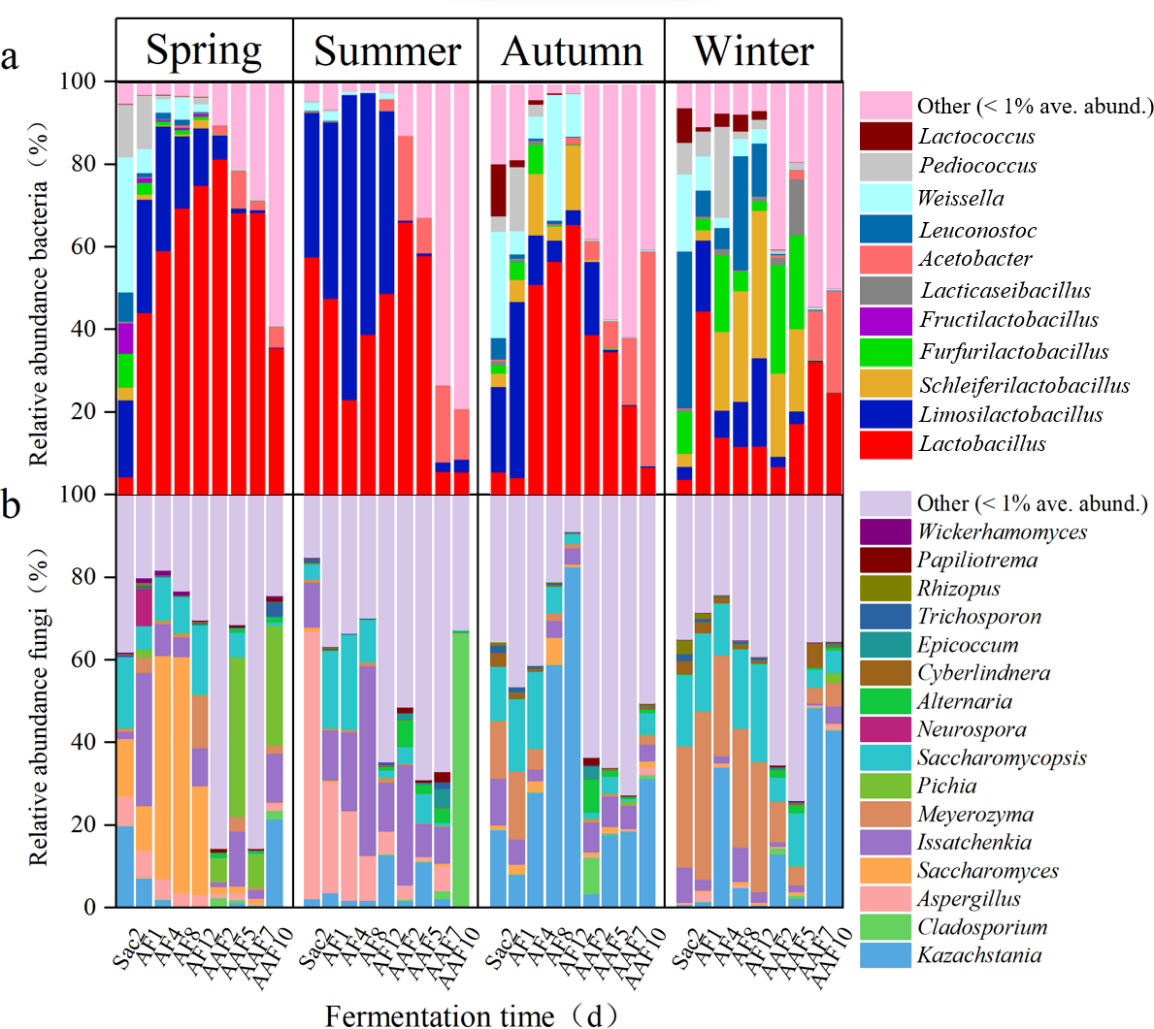
Relative abundance of bacteria (**a**) and fungi (**b**) throughout the SMV fermentation process in all four seasons, at the genus level. Only those genera with an average abundance above 1% in at least one season were indicated. Genera with abundance below 1% were combined and shown in the “others” category.

A total of 27 dominant genera (average relative abundance ≥1%) were shared in the SMV fermentation process throughout the seasonal cycle, with 11 bacteria and 16 fungi, respectively, while their relative abundances varied among seasons (Fig. 3). For bacterial taxa, except for Sac2 and AAF10, the relative abundance of *Lactobacillus* in spring was significantly higher than the other three seasons. The predominant microorganism of Sac2 in spring was *Weissella*. Compared with the other three seasons, the relative abundance of *Weissella*, *Lactococcus*, *Pediococcus*, *Leuconostoc*, *Lacticaseibacillus*, *Furfurilactobacillus*, and *Schleiferlactobacillus* significantly decreased during the whole fermentation stage in summer, indicating that they cannot adapt well to the high temperature in summer (30, 31). The abundance of *Lactobacillus* was the lowest in the later stages of AAF (AAF7 and AAF10) in summer. *Limosilactobacillus* was enriched during summer compared to the other seasons and became the predominant bacterial genus of AF4 and AF8 in summer, probably due to its tolerance to high temperatures. The relative abundance of Acetobacter increased significantly during the AAF stage and reached the highest at AAF10 in autumn (51.65%). Although the bacterial community structure varied seasonally, *Lactobacillus* and *Acetobacter* dominated across different seasons, contributing to the relatively stable quality of the vinegar.

As for fungal taxa, the microbiota structure showed a more diverse composition in the SMV fermentation process compared with the bacterial community, and the result is consistent with the alpha diversity analysis. At the genus level, *Kazachstania*, *Saccharomyces*, and *Pichia* dominated the Sac, AF, and AAF stages in spring, while *Aspergillus*, *Issatchenkia*, and *Cladosporium* prevailed in summer. In Wang’s work, it was observed that *Aspergillus* existed and increased throughout the entire AAF stage, but it maintained a low abundance overall (32). In this work, *Aspergillus* was enriched in summer and dominated at Sac1 and AF1 stages, demonstrating its ability to grow in a wide range of environmental conditions. The abundance of *Kazachstania* was lower in spring and summer, but it increased rapidly in autumn and became the dominant fungus throughout the entire autumn fermentation process. In winter, *Meyerozyma* and *Kazachstania* dominated in the AF and AAF stages, respectively. Compared to dominant bacteria, dominant fungi were more affected by season. Based on the shifts in the dominant species during different seasons, we speculate that fungi may be the main direct cause of quality differences in vinegar during different seasons.

Microbes with less relative abundance varied throughout the fermentation process and were exceedingly distinct in different seasons. For example, *Pichia*, the important fungus in spring, was virtually absent in the other three seasons. Although low in abundance, *Pichia* could influence the quality and flavors of the vinegar in significant measure (1). *Pichia manshurica*, isolated from Daqu, can significantly increase the production of acids and esters, endowing the vinegar with a pleasant fruity and floral flavor as well as a better aftertaste (1). Similarly, the spring season exhibited the highest abundance of *Wickerhamomyces* among the four seasons. *Wickerhamomyces* species are known for their ability to produce volatile compounds that contribute to the sensory properties of fermented foods (33). These results were indeed related to the better quality of vinegar produced in spring. In terms of the fermentation process, *Weissella* and *Leuconostoc* were the important genera in the Sac and AF stages in all four seasons but were not detected in the AAF stage. In addition, most of the microorganisms have been detected in previous studies on kinds of vinegar, while *Cyberlindnera*, *Neurospora*, and *Papiliotrema* were detected in cereal vinegar fermentation for the first time in this study (5, 34).

### Linear discriminant analysis effect size analysis of SMV fermentation

**Fig 4:**
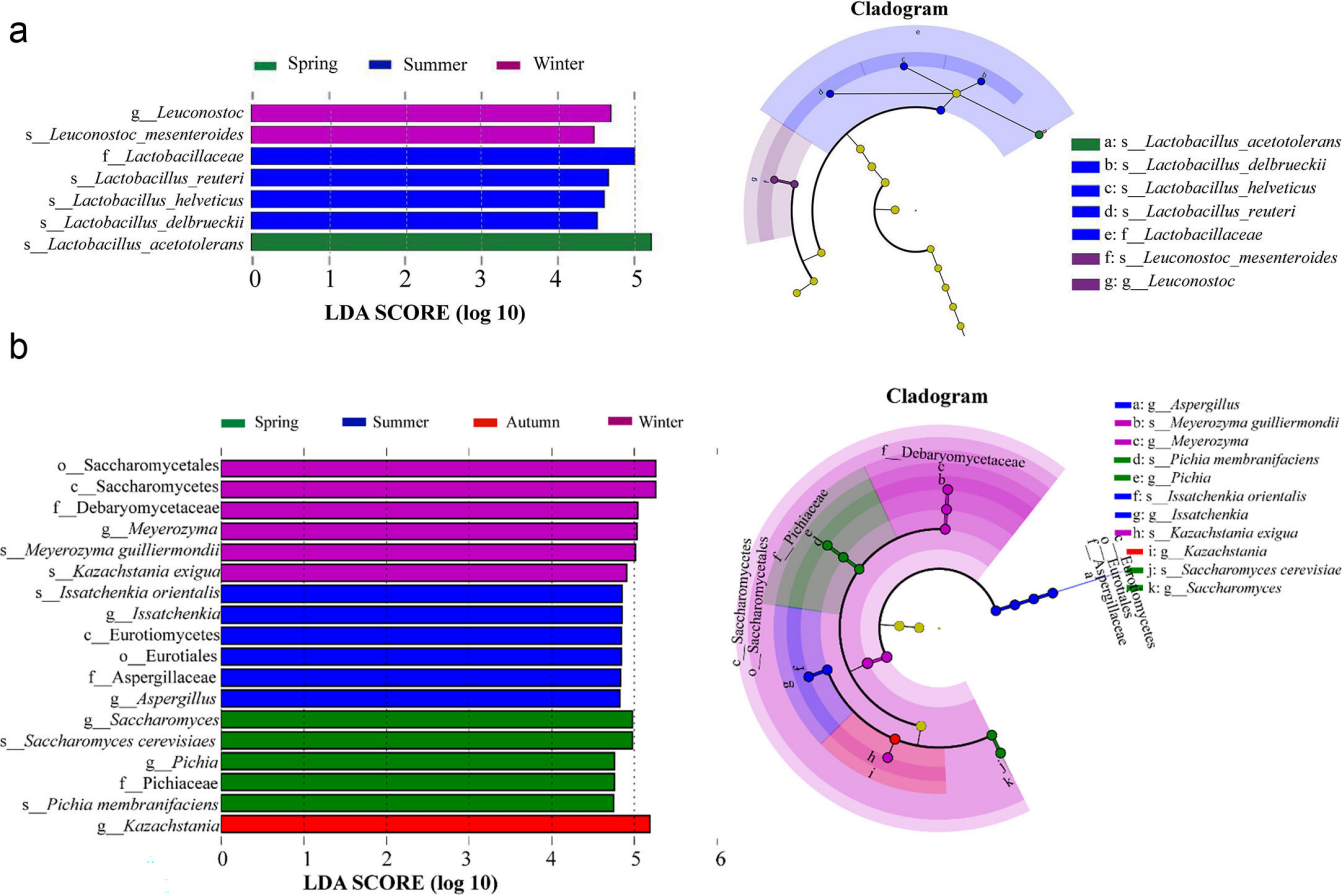
LEfSe analyses based on OTUs characterized the bacterial (**a**) and fungal (**b**) communities of the SMV fermentation process in different seasons.

LEfSe analysis was applied to identify the potential critical microorganisms in different seasons with an LDA score threshold ≥4.5 as the distinguishing feature. The results showed that a total of 25 microbial clades were obtained in all tested samples, comprising 7 bacteria and 18 fungi (Fig. 4a and b). The abundance of *Lactobacillus*, *Saccharomyces*, and *Pichia* in spring was significantly higher than in the other three seasons. *Aspergillus* and *Issatchenkia* were the characteristic microorganisms contributing to the samples fermented in summer. *Kazachstania* was the predominant microorganism in autumn, while *Leuconostoc*, *Mesenteroides*, and *Meyerozyma* were the significantly different genera in winter. These biomarkers were deduced to contribute to the distinctive microbiota composition in different seasons, thereby affecting the stability of SMV fermentation.

SMV is spontaneously driven by multi-species from a wide variety of resources, involving conventional matrix (sorghum, wheat bran, and rice husks), Daqu or vinegar *Pei* starter, and the open work environment, which account for the diversity of SMV microbiota. Although numerous microbes enter the naturally engineered vinegar ecosystem, the community-level microbial self-domestication process in the stressful ecosystem inhibits the growth of many spoilage microorganisms and maintains functional microbiota due to the highly selective micro-environment and operational conditions (35). Similar to previous studies on SMV (5), the predominant bacterial genera during semisolid-solid SMV fermentation in all seasons were lactic acid bacteria (LAB) and acetic acid bacteria (AAB). Specifically, *Lactobacillus (L.) acetotolerans* and *Acetobacter (A.) pasteurianus* were the dominant LAB and AAB, respectively (Fig. S3). Although some studies showed that *A. pasteurianus* was the primary bacterium for cereal vinegar fermentation (36, 37), in this study, *L. acetotolerans* was found to be the dominant bacterium and the abundance of *L. acetotolerans* in spring was significantly higher than those in the other three seasons.

In comparison with bacterial taxa, fungal taxa presented a much more diversiform during vinegar fermentation. *Saccharomyces* was identified as the predominant fungus in Shanxi mature vinegar and Zhenjiang aromatic vinegar, while *Alternaria* was the dominant fungus in Liangzhou fumigated vinegar (5, 34, 38). In our study, different dominant fungi were detected in four seasons from the fermentation process of SMV. *Saccharomyces* only showed higher abundance in spring, while *Issatchenkia* and *Aspergillus*, *Kazachstania*, and *Meyerozyma* were the fungi with higher relative abundances in summer, autumn, and winter, respectively. In this study, more abundant fungi were discovered, which can be attributed to the investigation conducted from a seasonal perspective. Certain fungal genera showed higher abundance in specific seasons, mainly influenced by seasonal changes in environmental factors such as temperature and humidity. In addition, the fungal community may play a more important role in the formation of varied components of SMV in different seasons than the bacterial community, while the composition of bacteria was more important for the stability of the vinegar fermentation. Dominant microbes, as mentioned above, could influence the fermentation microecosystem through their growth and metabolism, as the production of secondary metabolites is generally fed back to affect the microbiota profiles (39).

### Co-occurrence networks analysis of SMV fermentation

**Fig 5:**
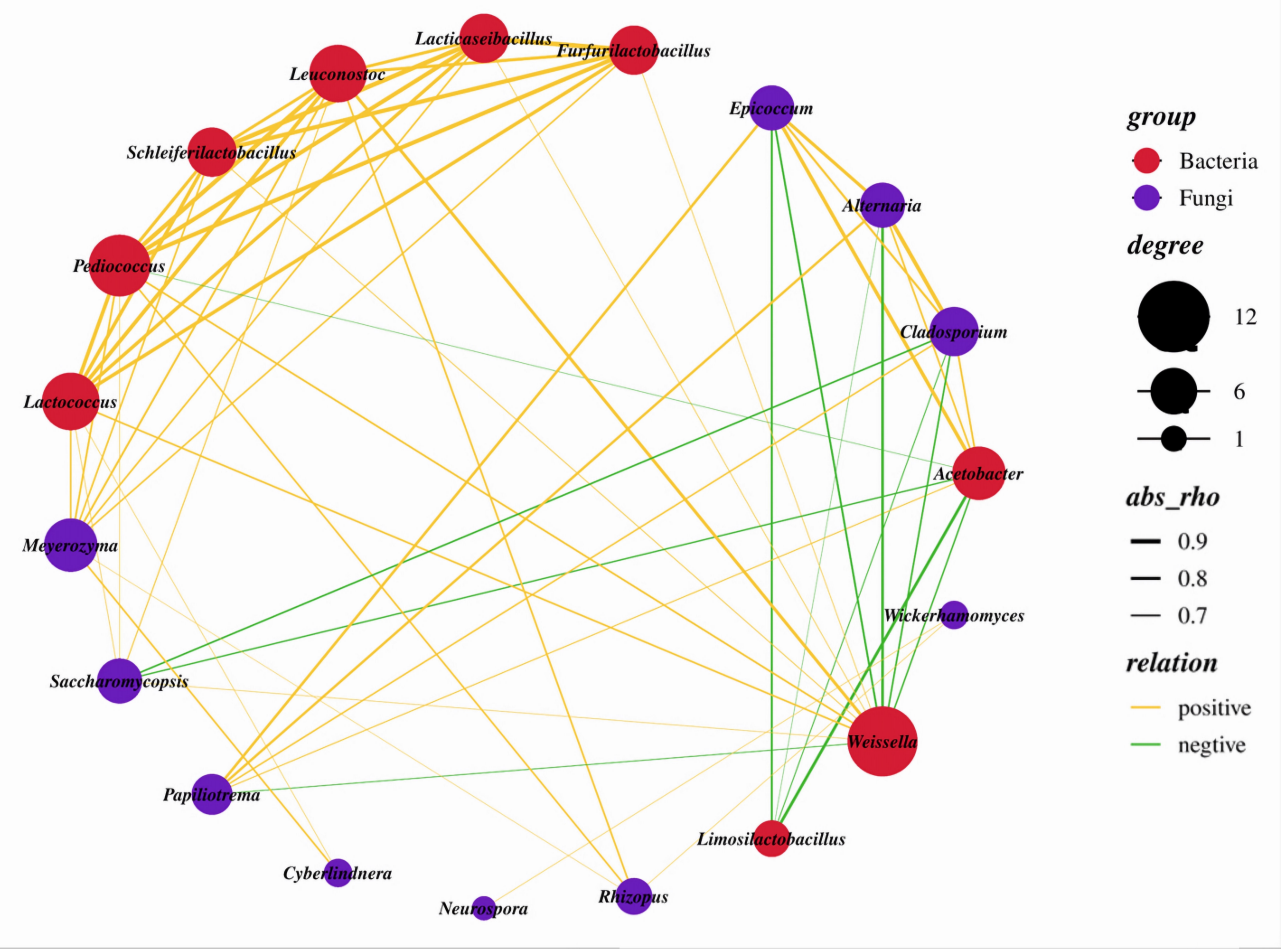
Co-occurrence network in the SMV fermentation. Only those nodes with average relative abundance above 1% in at least one season were included in the network analysis. A connection represented a statistically significant (*P* < 0.05) positive (Spearman’s r > 0.6) or negative (Spearman’s r < −0.6) correlation.

To investigate the interactions among microbiota in SMV fermentation micro-ecosystem at a large temporal scale, the co-occurrence and co-exclusion patterns were explored using Spearman’s correlation coefficient (Fig. 5). A total of 60 pairs of significant and strong correlations were identified from 27 genera taxa, including 9 bacterial genera and 10 fungal genera. The incidence of positive occurrence was higher than that of negative occurrence in the bacteria-fungi network, accounting for 80% (ratio of targeted edges to total edges) and 20%, respectively. The average degree (edges per node) was 3.158. There were eight hubs (highly connected genera, degree ≥5), including *Schleiferilactobacillus*, *Furfurilactobacillus*, *Lacticaseibacillus*, *Acetobacter*, *Leuconostoc*, *Weissella*, *Saccharomycopsis*, and *Papiliotrema*, indicating that they may play important roles in ecosystem stability. The negative correlations were mainly distributed in *Limosilactobacillus*, *Acetobacter*, *Weissella*, *Cladosporium*, *Saccharomycopsis*, and *Papiliotrema. Limosilactobacillus* was significantly and negatively correlated with *Acetobacter*, *Cladosporium*, *Alternaria*, and *Epicoccum*. Besides, *Acetobacter* showed negative correlations with *Weissella*, *Pediococcus*, and *Saccharomycopsis*, indicating that co-exclusion relationships existed between *Acetobacter* and these three genera. *Alternaria* showed a negative correlation with *Weissella*, which is extensively distributed in fermented products. The production of lactic acid by Weissella leads to a decrease in pH, which contributes to inhibiting the growth of pathogens and spoilage organisms (40). The fungal taxa including *Cladosporium*, *Alternaria*, *Epicoccum*, and *Papiliotrema* were positively correlated with each other, while *Cladosporium* showed a significant negative correlation with *Saccharomycopsis*.

Extensive interspecies interactions among microbiota are important in the vinegar microecosystem. There was an antagonistic relationship between *Acetobacter* and other bacteria, and the accumulated acetic acid produced by *Acetobacter* led to the decrease of other bacterial genera. Although significantly positive correlations between *Lactobacillus* and other genera have been reported in Fang et al.’s study (13), they are not observed in this study. Meanwhile, *Schleiferilactobacillus*, *Furfurilactobacillus,* and *Lacticaseibacillus* showed significantly positive relationships with other genera. It may be that the complicated interspecific interactions among the microbiota affect the dynamics and function of the microbiota (17, 41). Notably, *Cladosporium*, *Alternaria*, *Epicoccum*, and *Papiliotrema* were positively correlated with each other, indicating they shared common preferences in the vinegar brewing environment or formed a co-dependency pattern through metabolite exchanges (42). Overall, the co-occurrence network showed that the incidence of negative correlation was significantly lower than that of positive correlation, thus synergistic effect played a key role in community succession. The result is similar to the microbe interaction study in the fermentation of Zhejiang rosy vinegar (13). The inferred network structure represents a set of hypotheses as to potential interactions among genera (43), and these results can be further verified by microbial screening, identification, and bacteriostatic action by agar plate diffusion tests in the future. Seasons influenced the succession patterns of the microbial community in SMV fermentation. Therefore, further exploration should be conducted on the correlation between the microbial community and seasonal environmental factors, such as temperature, relative humidity, oxygen content, pH, and wild microorganisms, to ensure the stability of SMV quality. In addition, due to higher temperatures, dominant microorganisms such as *Acetobacter* and *Lactobacillus* decreased in summer. Screening or modifying this group of bacteria to enhance their tolerance to high fermentation temperature is also an important approach to improve industrial SMV fermentation (44).

### Conclusion

In conclusion, a high-throughput sequencing technique was used to describe the seasonal assembly and succession of microbiota communities during the SMV semisolid-solid fermentation in this study. The impact of seasons on potential core microorganisms and the co-occurrence between microorganisms were further investigated. This work provides insights at a temporal scale for understanding the effects of seasonal changes on microbiota assembly and offers new guidance for the process control of grain vinegar brewing, especially in high-temperature periods.

### Research highlights

1. *Lactobacillus* and *Acetobacter* dominated across different seasons.
2. Dominant fungi were more diverse than bacteria throughout the seasons.
3. The unique fungal structure may be an important reason for the low quality of summer vinegar brewing.

## SUPPLEMENTAL MATERIAL

Figure S1 to S3 spectrum.00231-24-s0001.docx Relative abundance of bacteria and fungi.

Table S1 spectrum.00231-24-s0002.xlsx The sequencing quality control data of Ion S5TM XL platform for 36 samples.

Table S2 spectrum.00231-24-s0003.xlsx The alpha diversity index of bacteria and fungi.

ASM does not own the copyrights to Supplemental Material that may be linked to, or accessed through, an article. The authors have granted ASM a non-exclusive, world-wide license to publish the Supplemental Material files. Please contact the corresponding author directly for reuse.
