# Supplementary material for: Dynamics and biodiversity of microbial community among seasons in Shanxi mature vinegar fermentation by semisolid-solid process
Source: Microbiol Spectr. 2024 Nov 13;12(12):e00231-24. doi: 10.1128/spectrum.00231-24 (PMC11619342; doi:10.1128/spectrum.00231-24)
Supplement: Figure S1 to S3 — Relative abundance of bacteria and fungi. [file spectrum.00231-24-s0001.docx]

**

Fig. S1.** Rarefaction curves analysis of bacteria (a) and fungi (b) throughout the SMV fermentation process in all four seasons, based on the observed species.

**
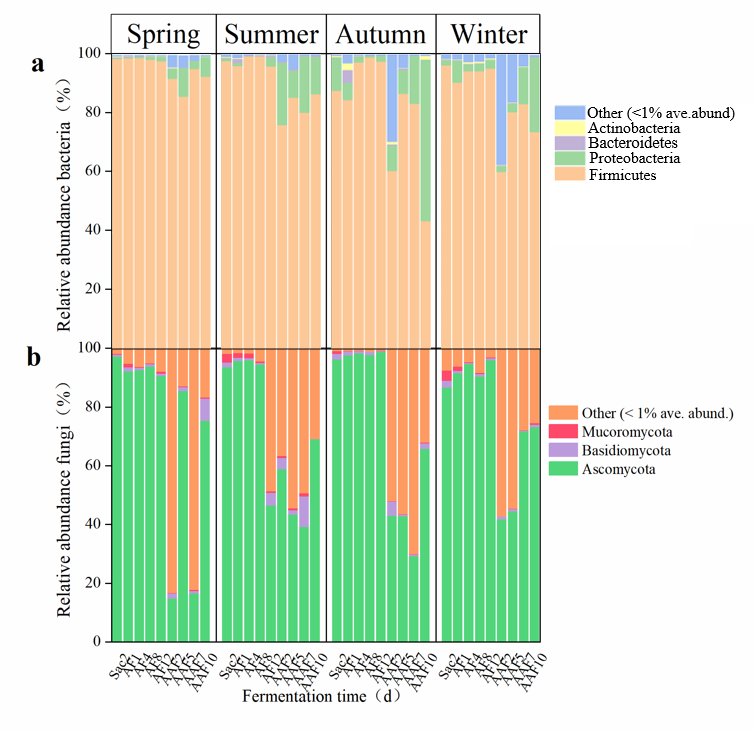
Fig. S2.** At the phylum level, relative abundance of bacteria (a) and fungi (b) throughout the fermentation process in all four seasons. Only those phyla that had an average abundance greater than 1% in at least one season were indicated. Phyla with less than 1% abundance were combined and shown in the “others” category.

**
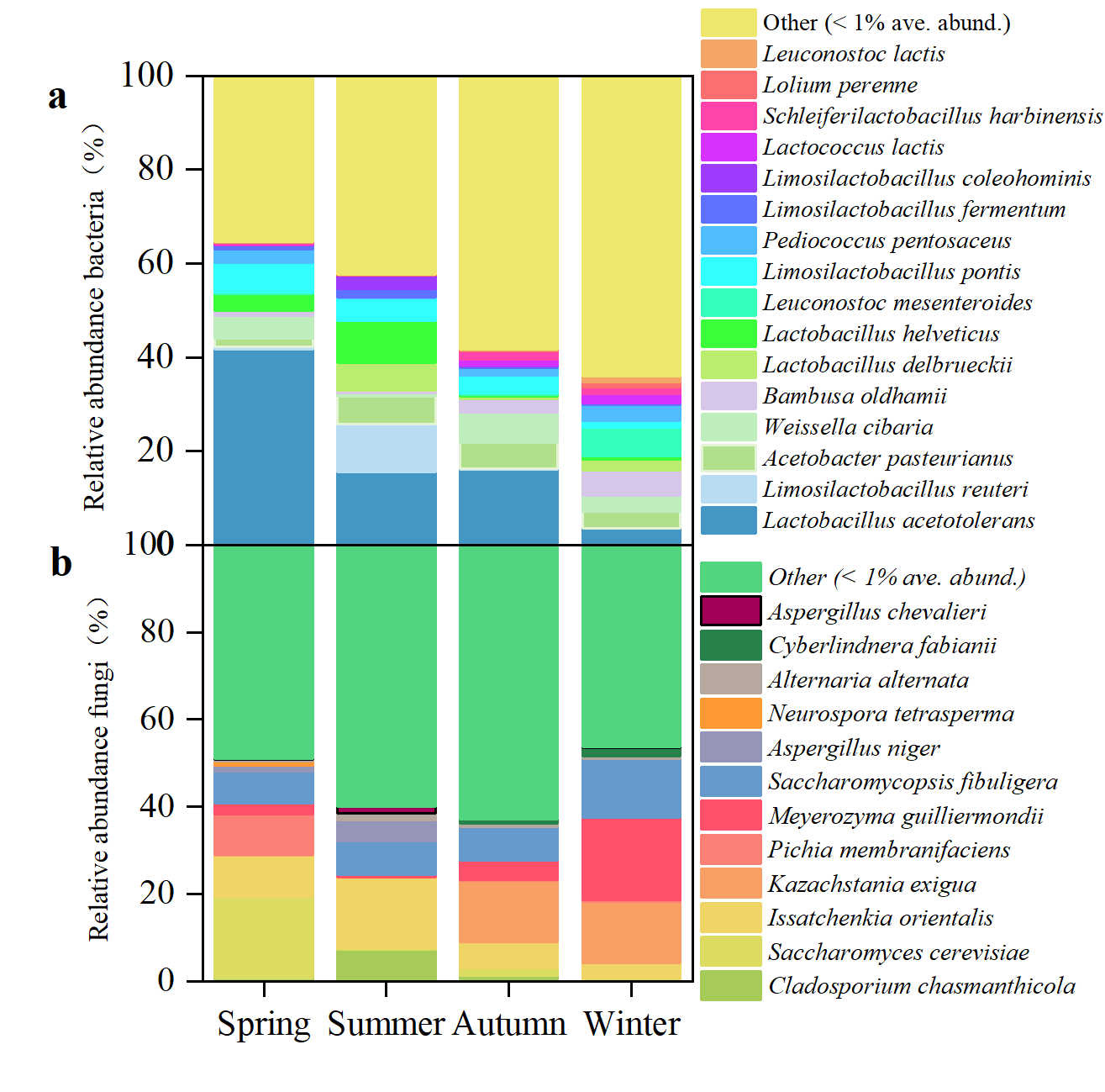
**

**Fig. S3.** At the species level, relative abundance of bacteria (a) and fungi (b) in all four seasons. Only those species that had an average abundance greater than 1% in at least one season were indicated. Species with less than 1% abundance were combined and shown in the “others” category.
